# Surgery

**Published:** 1858-03

**Authors:** 


### SURGERY.

1. ON THE EMPLOYMENT OF COLLODION IN ORCHITIS. By *Dr. Lossetti*.—Having treated sixty-four cases of gonorrhœal orchitis in the Maggiore Hospital, Milan, solely by means of collodion, Dr. Lossetti comes to the following conclusions: 1. Orchitis, epididymitis, or inflammation of the spermatic cord, even when intense, arising from blennorrhagia, yields much more readily to the application of collodion than to the ordinary antiphlogistic treatment actively employed. 2. The most efficacious means of obtaining certain and rapid recovery is the application of collodion (the author prefers to pure collodion a mixture of one part of castor oil and twenty-four parts of collodion) twice a day, until the cure is completed, applying it oftener, however, in cases of extreme gravity. 3. Under the use of the collodion, the indurations and enlargements which remain after the relief of the inflammatory action will be dispersed without the aid of any other means. 4. By means of its application, the erysipelas of the scrotum that sometimes complicates the orchitis becomes resolved, and the cellular infiltration that almost always accompanies or succeeds it disappears. 5. The treatment required besides, consists only in the observance of a low diet, rest, and cooling drinks. 6. This mode of treatment is more economical than the antiphlogistic mode, by reason of the shorter time it requires, (according to the author's cases, eight days as compared to fourteen by the ordinary treatment,) and the saving of expensive remedies, as leeches, etc. 7. The treatment is also to be preferred, since the digestive organs are not disturbed by active purgation, and the general powers are not enfeebled by this and the abstraction of blood. 8. The sensation of burning produced by the application of the collodion never amounts to the extent of acting as a contra-indication. It becomes violent, and even insupportable, only when some minute lesions of continuity of the skin of the scrotum are present; and these only require to be protected by cerate or court-plaster, to enable the patient to bear the application.—*Omodei Annali Universali. Med. Times and Gazette*, Jan. 16, 1858.

2. RULES RESPECTING THE TREATMENT OF PRIMARY SYPHILIS.—It seems to be now pretty generally acknowledged, in hospital practice, that mercury should be given only in those cases in which the chancre presents marked induration, and that in all others secondary symptoms should be waited for before having recourse to specific treatment. In a large majority of sores not attended by induration, no constitutional phenomena will follow; and to discriminate between those likely to be so followed and the harmless class is admitted to be impossible. There is, therefore, no alternative, except we would give mercury very often unnecessarily, but to wait in these cases until the real nature of the affection shall have been made manifest. In the non-indurated class local stimulants, as sulphate of copper, lunar caustic, or the acid nitrate of mercury, are the old and still favorite remedies. If the chancre be seen within a week of its origin, whether induration have already commenced or not, we believe most surgeons would destroy it freely either by nitric acid or some other caustic.—*Ibid.*

3. SYPHILITIC INFLAMMATION OF THE RETINA.—The revelations of the ophthalmoscope bid fair to add a peculiar form of retinitis to the acknowledged *rôle* of symptoms due to constitutional syphilis. A fortnight ago we noticed a very interesting case, in which lymph had been seen deposited on the retina of an infant, the subject of hereditary syphilis. A few days afterwards Mr. Critchett admitted a second case, in which a girl, whose history and appearance led to the belief that she was the subject of the same kind of taint, was losing sight in both eyes, from the punctate effusion of lymph on the retina. Two other cases are attending Mr. Critchett's clinique, in which, in connection with acquired syphilis, retinitis with effusion has occurred. In one the effusion is in the form of isolated white dots; but in the other the whole visible extent of retina is cloudy and opaque, the optic nerve itself being but dimly seen. It is worthy of note, that in neither of these cases has there been any iritis or affection of the anterior parts of the globe.—*Ibid.*, January 2, 1858.

4. OBSERVATIONS ON THE DIAGNOSIS OF HYDROCELE. By *Professor Nélaton.*—M. Nélaton recently addressed the following observations to his clinical class:—In some cases the diagnosis of tumors of the testis presents almost insurmountable difficulties. The slightest circumstances then acquire the highest importance, and it becomes of importance that we should not attribute to any sign a degree of confidence that it does not deserve.

*The Transparency of Hydrocele.*—In order that this should be effectually exhibited, we should take care that the patient is not exposed to broad daylight. Another point just as indispensable is often neglected. When the surgeon commences his exploration, the sudden impression of cold causes a contraction of the dartos, and however slightly the size of the tumor may allow of its rising toward the ring, to that extent the scrotum becomes relaxed, wrinkled, and increased in thickness. This is one of the impediments to transparency, but there is another of much more importance. This ascending movement, by bringing the tumor nearer the axis of the body, prevents the light from falling perpendicularly on its surface; and the rays which fall under too oblique an incidence do not penetrate the tumor, and consequently cannot exhibit its transparency. It becomes necessary, therefore, to isolate the tumor as much as possible, to, so to speak, enucleate it, by taking hold of it near the ring and pressing it forward, separating it as much as possible from the other parts of the body. In this way the wrinkles of the scrotum also disappear, and the stretched skin diminishes in thickness. Transparency may now be easily shown, by placing the light very close, (two or three centimetres,) and on a transverse axis passing through the middle of the tumor, so that the rays may traverse it unreflected. This precaution, of no account in simple cases in which error is well nigh impossible, is indispensable in those in which the surgeon remains undecided as to the nature of the disease, precisely because he cannot establish the transparency. M. Nélaton has met with many cases in which the surgeon, from want of being aware of these facts, has mistaken hydrocele for far more important affections of the testis.

*Specific Gravity.*—Most surgeons attach very great importance to this sign, but it will be easy to show that it does not deserve the confidence placcd in it. The hand, raising the tumor, has to estimate the weight of the entire mass; and these tumors, when even of the same nature, possess very different amounts of movability, accordingly as they are attached by pedicles, letting the hand pass on all sides, or are bound down by the contraction of the cremaster, dartos, and scrotum. Moreover, we have not to appreciate the absolute weight of the tumor, but its relative specific weight, as compared with that of another mass of a different size—a mass not now at hand capable of being compared, but estimated at some former period, under similar unfavorable conditions, its specific gravity being retained in the memory.

Let a piece of gold of a certain volume be placed in the hand of an individual, and a week afterward give him a piece of copper of the same volume, desiring him to appreciate the relative specific weight of the two metals. Certainly he will not do this readily, and the task will become far more difficult if we present to him pieces of different size. And yet the specific weight of gold is more than double that of copper. What must happen, then, when we have to compare with each other, under the above-mentioned unfavorable conditions, masses of a specific weight so slightly differing as hydrocele and the other diseases of the testis? For, in fact, the difference of the specific weight of the liquid of hydrocele, and of that of the matters constituting other diseases of the testis, is almost insignificant. Of this we may be easily convinced by examining the figures M. Nélaton has obtained by comparing the weight of various tumors with the weight of the same volume of water.

A hydatid cyst of the testis, weighing 466 grammes in the air, displaced 450 of water, giving a difference of less than $\frac{1}{30}$. An encephaloid tumor gave a difference of $\frac{1}{40}$, and a cancerous sarcocele gave a difference of about $\frac{1}{13}$. Now it is evident that a difference even of $\frac{1}{12}$ is quite inappreciable in the conditions under which the surgeon operates, while the density of the serosity of hydrocele is somewhat greater than that of water, and in the experiments the weight of the tumors are compared with that of water, without taking into account the addition of tissues which exist in the living subject. But these tissues, viz., the testis and its coverings, are nearly of the same density as encephaloid matter. It may be then said that the sign drawn from the specific gravity of tumor of the testis is positively of no value. It is one of those signs invented in the study, and has continued to be handed down to us because no one has thought of examining into its exactitude.—*British and Foreign Med.-Chir. Review*, January, 1858; from *Gaz. des Hôpitaux*, No. 124.

5. REDUCTION OF HERNIA BY COMPRESSION.—In the employment of the taxis too much weight is sometimes laid upon the energy of the efforts and the violence exerted upon the herniated parts. A man, aged fifty-four, was brought into M. Nélaton's wards, having a large irreducible inguinal hernia. Many attempts at reduction had been made without success, when M. Nélaton employed compression in the following manner:—A strip of gutta-percha was placed over the upper part of the thighs, below the scrotum, and kept immoveably in this position by means of bandages; a canvas bag filled with three or four pounds of sand was then applied over the tumor; this produced a uniform pressure, which was easily borne, and the mass diminished in size. The taxis was practiced from time to time until the whole was returned.—*Medical Times and Gazette*, January 23, 1858; from *Bull. de Thérap.*, December, 1857.

6. ACUTE GLAUCOMA CURED BY A NEW OPERATION. (Under the care of *Mr. Critchett.*)—An operation has recently been performed by the above gentleman, so novel in its character, so simple in its execution, and, as the subsequent history of the case proved, so successful in its result, that we are desirous of taking this early opportunity of bringing a few leading particulars of the case before the notice of the profession.

The subject of the operation we are about to describe was a working engineer, aged thirty-four. A few days previously he was attacked with sudden and severe inflammation of the globe, with intense pain. The pupil was fixed and widely dilated; the lens looked dull, and the sight was so dim that there was merely perception of light. The other eye had been destroyed by an accident about five years ago. Mr. Critchett introduced a broad needle through the cornea, close to its junction with the sclerotic into the anterior chamber; he then drew out a portion of the iris and cut it off, leaving at the same time a small portion in the wound. The immediate effect of this proceeding was entirely to relieve the pain, and we learned that the sight gradually improved and the pupil recovered its mobility. In about a fortnight the man was able to read moderately sized print.

Mr. Critchett remarked that he considered this a case of acute glaucoma, and the object of the operation was to relieve the distension of the globe, and, by leaving a portion of iris in the wound, to prevent for a time an undue reaccumulation of fluid. He had recently adopted this proceeding four times, in similar cases, with the best results, and he felt that a disease which he, in common with all ophthalmic surgeons, had hitherto regarded as fatal to sight, was now brought within the control of art by a slight and safe operation.—*Lancet*, January 30, 1858.

